# Women survivors of intimate partner violence talk about using e-health during pregnancy: a focus group study

**DOI:** 10.1186/s12905-022-01669-2

**Published:** 2022-03-31

**Authors:** Rodrigo Fernández López, Sabina de-León-de-León, Stella Martin-de-las-Heras, Juan Carlos Torres Cantero, Jesús L. Megías, Antonella Ludmila Zapata-Calvente

**Affiliations:** 1 Department of Preventive Medicine and Public Health, University of Granada, Granada, Spain; 2 Brain and Behavior Research Center (CIMCYC), University of Granada, Campus de Cartuja S/N, 18011 Granada, Spain; 3 Department of Forensic Medicine, University of Málaga, Málaga, Spain; 4 Malaga Biomedical Research Institute (IBIMA), University of Málaga, Málaga, Spain; 5 Department of Software Engineering, University of Granada, ETSIIT, Granada, Spain; 6 Department of Social Psychology, University of Granada, Granada, Spain

**Keywords:** Intimate partner violence, Pregnancy, eHealth, Video counseling, Safety plan, Healthcare providers

## Abstract

**Background:**

Pregnancy is a period of particular vulnerability to experience intimate partner violence against women (IPVAW). eHealth strategies have been implemented to identify women exposed to IPVAW and to combat the abuse and empower them, but there is a lack of evidence on the use of these strategies among pregnant women. This work aims to identify the needs, concerns and preferences of survivors about the use of eHealth strategies to counsel and empower pregnant victims of IPVAW in antenatal care.

**Methods:**

A focus group of six IPVAW survivors who had been pregnant was conducted and open questions about the use of eHealth strategies were asked. The session was recorded, transcribed and thematically analyzed. We identified three main themes: needs and worries of pregnant women experiencing IPVAW, key aspects of video counseling sessions and usefulness of safety planning apps.

**Results:**

Women highlighted the relevant role of healthcare professionals—especially midwives—in the identification of IPVAW and the wellbeing of their children as one of the main concerns. They perceived video counseling and safety planning apps as valuable resources. The preferred contents for a video counseling intervention were awareness-raising of the situation, self-esteem and legal advice. They also proposed safety and pregnant-related aspects that should be taken into account in the design of the video counseling sessions and the safety planning app.

**Conclusions:**

Video counseling sessions and safety planning apps are potentially useful tools to counsel and empower women who experience IPVAW during pregnancy. Midwives play a key role in this endeavor.

## Background

Intimate Partner Violence Against Women (IPVAW) is a global health issue that affects women and their children. According to the World Health Organization, IPVAW is defined by any act of physical, sexual and/or emotional abuse perpetrated against a woman by a current or former intimate male partner [1]. Global prevalence of IPVAW estimates show that around 30% of women experience some kind of violence from their partner in their lifetime [1]. In the European Union (EU), IPVAW has a prevalence of 22% for physical violence, while that of psychological violence is estimated to be around 43% [2]. More specifically, in Spain, the general prevalence of IPVAW is 24.8%, of which 16% is estimated to be physical and 21.1% is estimated to be psychological [3].

It has been established that IPVAW has many negative consequences for victims. Women who experience IPVAW are more likely to have various physiological conditions affecting the central nervous system, the cardiovascular system or the reproductive system, among others [4]. Physical abuse results in severe injuries from blunt trauma, particularly in the neck, face and head [5]. Severe IPVAW exposure can lead to chronic health problems, disability and death [4]. Regarding mental health, exposure to IPVAW is associated with depression [6], post-traumatic stress disorders [7], anxiety disorders [8] and substance abuse [4].

Pregnancy is a period of vulnerability when considering IPVAW. Multi-site studies estimate that the prevalence of IPVAW during pregnancy varies between countries and ranges from 1 to 28% [9]. Looking for a more detailed description of the problem, a meta-analysis of 92 independent studies concluded that the average reported prevalence of emotional abuse during pregnancy was 28.4% (13.8% for physical abuse and 8.0% for sexual abuse) [10]. Specific consequences associated with IPVAW during pregnancy have also been explored and a variety of physical and mental health-related issues in victims and their children have been found. Pregnant women who experience IPVAW are more likely to have urinary tract and vaginal infections [11], post-natal depression symptoms [12] and higher levels of anxiety [13]. Furthermore, they are more likely to avoid breastfeeding [14] and to have poor antenatal care attendance [15]. In addition, IPVAW during pregnancy can cause a wide range of problems in children, ranging from low birth weight and premature birth to fetal loss and perinatal and neonatal death [16]. Moreover, it is associated with future development of behavioral problems in children and adolescents [17].

Efforts are made to reduce IPVAW exposure and to palliate its negative consequences in the health care system. However, there are several barriers for women who experience IPVAW to find help or report the violence in this environment. These barriers can be personal and related to feelings of shame and fear or due to lack of awareness of the situation. Alternatively, some of these barriers can be structural and related to the nature of the health care system itself [18].

An alternative way of palliating the negative effects of the barriers that IPVAW victims encounter when seeking help and reporting violence can be to implement electronic health (i.e., eHealth) strategies. eHealth refers to the use of information and communication technologies for health issues and is a broad concept that comprises various areas such as telehealth and mobile health [19]. eHealth interventions have the potential to provide more flexibility and safer spaces than face-to-face interventions, making it particularly useful for screening violence, empowering women and reducing exposure to IPVAW [20].

eHealth screening tools have proven to be an effective alternative way of detecting violence in victims [21, 22]. Several eHealth interventions based on safety decision-making [23–25] and psychological wellbeing [26, 27] have also shown effectiveness in reducing IPVAW exposure and its psychological consequences. In contrast, some authors highlight that the evidence in favor of these type of interventions in victims of IPVAW is not consistent and flawed with methodological issues, stating that more research is needed to reach a conclusion [28].

A possible reason for such mixed results is that current eHealth interventions are not fully adapted to women’s situation and moment of life [29]. For the last 20 years, there has been growing interest in including the public and patients’ opinions into health services and the work of health professionals [30]. Patient and Public Involvement (PPI) is becoming an important aspect when developing new health care interventions, because listening to patients’ voices can lead to better designed services and more positive experiences for users [30]. Thus, in order to design effective eHealth interventions for pregnant women, it is important to know the views and opinions of women who have been pregnant and survived IPVAW. This is likely to lead to eHealth strategies that are be better suited to the needs of pregnant women who experience IPVAW, thus avoiding potential barriers and optimizing the possible beneficial effects of interventions. The objective of the present research was to identify needs, concerns and preferences of survivor victims of IPAW about eHealth strategies to counsel and empower pregnant victims of IPVAW in antenatal care.

## Method

### Design

This work consisted of a qualitative study with a phenomenological approach [31] to explore the views and opinions of women who have been victims of IPVAW about the use of eHealth interventions for pregnant women who suffer IPVAW.

### Study setting and participants

Participants were recruited through a purposive sampling considering as inclusion criteria: 1. Adult woman; 2. A previous victim of any type of IPVAW; 3. Previous pregnancy; 4. Fluent Spanish speaker. The women were recruited through a municipal women’s information center, a specialized unit for women and, in particular, for victims of gender-based violence in the south of Spain. Two professional psychologists offered women who met inclusion criteria to participate in qualitative interviews after briefly explaining the study. Women who agreed to participate were contacted by a researcher to provide further details about the study and to obtain their informed consent. Subsequently, the qualitative interview was led by a researcher and an observer and conducted in September 2020.

#### Demographics

Table 1 summarizes the demographic data of participants. They were all from Spain, had 1 to 3 children and had experienced IPVAW in the past. Their level of education ranged from high school graduate (N = 2) to university studies (N = 4). Most of the women were in their forties (N = 5) while one woman was in her thirties (N = 1). Regarding employment status, some women were unemployed (N = 2) while the rest had a job (N = 4). Most participants were divorced (N = 3), but some were single (N = 2) or separated (N = 1).

**Table 1:** Demographic data of focus group participants

Participant number	Age	Citizenship	# Children	Marital status	Job	Education
1	47	Spanish	1	Divorced	Cleaner	Master’s Degree University Degree
2	48	Spanish	3	Divorced	Hosting international students at home	Nurse aide University studies (not completed)
3	47	Spanish	1	Divorced	Bookstore clerk	University studies (not completed)
4	45	Spanish	2	Single	Unemployed	Master’s Degree
5	40	Spanish	1	Single	Preparing exams for public job at the university	University studies
6	38	Spanish	2	Separated	Editor	Master’s Degree

### Data collection

Semi-structured interviews were conducted through a focus group of women who had been victims of IPVAW and had been pregnant in the past. Focus group discussions can provide not only relevant information about how best to implement new health interventions but also collective views and experiences on sensitive topics, such as IPVAW [32].

The interviewers prepared an interview guide with open questions about the thoughts and opinions of participants regarding several aspects of eHealth strategies (see Table 2). The interview guide and the focus group were developed following recommendations from specialized authors [33], progressively moving from initial general questions to more specific ones. The eHealth strategies discussed included the feasibility of a video counseling session directed to IPVAW prevention and the use of a safety planning app. Questions were arranged into three main themes that served as a reference for the qualitative analysis: (1) Support needs, preferences and concerns of pregnant women who experience IPV; (2) Key aspects that should be taken into account when conducting psychological video counseling focused on pregnant women who experience IPVAW; and (3) The opinion of survivors about the use of a safety app focused on pregnant women who experience IPVAW. In the latter theme, participants were asked their opinion about including typical features of safety apps, such as emergency contacts, peer stories, mood rating, information about the approached topic and safety planning.

**Table 2:** Interview questions for the focus group

**1. Understanding the support needs, preferences and concerns of pregnant women who experience IPVAW**
1.1. What was the most important help or support you received that you can highlight as especially useful for you?
1.2. What kind of help or support would you have liked to receive at that time or what kind of help do you think a pregnant woman who experiences intimate partner violence would like to receive?
1.3. What do you think are the main concerns of a pregnant woman when she is in a violent relationship? [for example, consequences of leaving her partner? concern about her pregnancy?]
**2. Key aspects that should be taken into account when conducting video counseling** 2.1 What content or what topics do you think are most important for working with a pregnant woman who is experiencing intimate partner violence in the 6 video counseling sessions that we provide to them? 2.2 What is the first thing that comes to mind or what do you think when you imagine the possibility of talking to a counselor through a video call? 2.3 What specific things could the counselor do to make you feel safe during the video counseling sessions? 2.4 When and where do you imagine yourself receiving those video counseling sessions? (i.e., at home, away from home…) 2.5 Is there anything you think you could not talk about if the counseling was done through video calls?
**3. Safety plan: opinion about the use of the app** 3.1. In your experience, what are the most important things for a woman to feel safe in an abusive relationship? 3.2. Do you think that we should take something specific into account when addressing safety with women who are pregnant and experience intimate partner violence? 3.3. Can an app like this increase the safety of women in this situation? We are now going to look at each feature separately (i.e., Contacts, Peer stories, Mood rating, Information on how IPVAW can affect pregnancy, Safety plan) and I will ask you to answer the following questions: (i) Any thoughts on this feature? (ii) Is there anything you would change or add? (iii) Can you imagine using this function of the app? (iv) After seeing the entire app, what do you think about using an app like this to develop women’s safety plans?

The focus group session lasted for 2 h. To obtain the data, the focus group audio was recorded with the consent of participants and then transcribed into text. This study was approved by the Andalusian Research Ethics Committee (study code 881648).

### Data analysis

Qualitative data were analyzed using thematic analysis [34]. The analysis was organized using the three main topics addressed in the script as a reference, although the organization was not strictly set and variations were included according to the participants’ responses. Transcripts of the focus groups were coded using ATLAS.ti 8 software for Windows 10. The three main themes were clustered into categories according to the semi-structured questions that were used during the focus groups. Four levels of coding were performed for the three main themes of the study. At the first level, the content of the transcripts was coded trying to stay as close to the data as possible, capturing in a few words the meaning of the data with little to no abstraction. At the second level, codes were developed to represent the meaning of women’s words. At the third level, the codes were collaboratively grouped into categories that fit the themes and sub-themes defined by the questions with the aim of drawing more abstract and shared meaning. Finally, at the fourth level, categories were integrated into the greater themes and sub-themes according to the relationship between those categories. See Table 3 for a coding tree.

**Table 3:** Coding tree: needs and preferences of pregnant women about using eHealth strategies for IPVAW

Theme	Sub-theme	Categories
Needs and concerns of pregnant women in an IPVAW situation	Support	*Role of health care professionals* *Psychological and emotional support*
	Concerns	*Children and the unborn baby* *Barriers for leaving the relationship*
Video counseling intervention: key aspects	Contents	*Awareness* *Self-esteem and fears* *Legal advice*
	Video-based counseling	*Feasibility* *Safety* *Barriers*
Contributions for safety planning apps	Safety using the app	*Password and masking* *“Quick leave” button*
	App resources	*Contacts* *Peer stories* *Mood rating* *Information on how IPVAW can affect pregnancy* *Safety plan*

## Results

As a result of the thematic analysis, three main themes and six sub-themes were identified: (1) Needs and concerns of pregnant women in an IPVAW situation, (2) Video counseling intervention: key aspects and (3) Contributions for safety planning apps.

### Needs and concerns of pregnant women in an IPVAW situation

In this first section, a general discussion was raised about the situation of pregnant women that experience IPVAW. Participants were asked about key aspects that might influence a victim during that period.

#### Support

Participants in the focus group found it particularly relevant to address the support that victims of IPVAW get and what can be done to improve it.

- Role of health care professionals

One of the main themes that came up in discussions about support was the role that health care professionals can play. Women pointed out that midwives and gynecologists are visited repeatedly throughout pregnancy, which is why they considered that such professionals have a great opportunity to discover the abuse. They considered that administering a questionnaire or simply asking the woman "*if she is happy with her situation, or if she is experiencing it well*", as Woman 2 pointed out, could be a good start.

Woman 5 recalled how her own midwife was the one who realized that something was wrong in her relationship, and emphasized how these professionals can help a lot to detect these kinds of situations in pregnant women.

Woman 5: *“my midwife, who was the only person who probably realized and, although I am somebody who doesn’t talk, I don’t talk much, she started to ask me questions I didn’t understand. And I never managed to figure how she realized some situations, as I don’t talk. But it’s true that she inquired and gradually started to ask more. She didn’t find out everything, obviously, but these people can help a lot.”*

Regarding this topic, another proposal made by the women was to establish a code that a woman can use to tell her midwife or physician that she is in a violent relationship. In Spain, due to the COVID-19 situation, a strategy has been implemented in pharmacies that consists of a code ("mask-19"), which a woman in a situation of abuse can request and triggers the activation of the necessary resources to help her.

Woman 1: “*perhaps now with the COVID situation, if you are abused, isn’t there a code you can use when you go to the pharmacy? I think there is something like that with the family doctor.”*

The fact of involving health care professionals is also related to the proposal of one of the women, who mentioned how important it was for the person who found out about the violence to be someone outside her social network, since she was worried about the safety of her loved ones and it was difficult for her to envisage involving her friends or family in her problem:

Woman 2: *“Because of this, the help of somebody who is not from your family or a friend is perhaps more positive, more real or better than somebody who is emotionally close to you and may worry (…) I thought, “I’m going to tell my brother because this has to end” but never told him, ever, for fear of the possible consequences. I thought… imagine that I end up harming the people that I love.”*

- Psychological and emotional support

Psychological support seems to be one of the main needs of a pregnant woman who is in a situation of IPVAW. Some women agreed that psychological help was crucial during their process of ending the relationship. For example, Woman 2 doubted that a counselor could help her at first, but eventually concluded that it was a good decision:

Woman 2: *“For me, for example, the counselor (..) was the solution to my head. I mean, it was the best thing I could do, it’s clearly the best help I’ve had during the 3 years of this situation. It was the best, I never thought it would do me so much good or that it was going to put me in such a position… In fact, when I went at first I thought, “This is not going to help me at all; I’m sure of what…” That’s why I think psychological help is super important.”*

Unlike her, Woman 6 said that she was sure that this type of help was the one she needed from the beginning:

Woman 6: *“I knew the first thing I needed was a counselor; the lawyer would come afterwards. But the counselor was first. Because I needed it.”*

#### Concerns

Different concerns that pregnant women might have when experiencing IPVAW and trying to end the relationship were widely discussed in the focus group. The women were worried about the situation other women might face when thinking about the children and the consequences of ending the relationship.

- Children and the unborn baby

Both the unborn baby and children are of great concern to pregnant women who experience IPVAW. The women explained how they became aware that their feeling of vulnerability, helplessness and danger affected not only them but also the children.

Woman 1: *“when you are pregnant you are more vulnerable, you are more afraid of… not only for you but for the baby you are carrying. (…) Well, the first thing that happens is that you feel twice as frightened or you feel very vulnerable because you are carrying a baby and I think on top of it they know it and take advantage of that.”*

The perceptions they may have about the aggressive behaviors of their partners and the actions they can take to increase their safety become more relevant when a baby is on the way. Both the wellbeing of their children and the need to be well in order to continue looking after their children becomes more latent.

Woman 2: *“I left to protect my daughters, not only to protect [my children] but to protect them from losing me. I mean, in principle he was not going to hurt them because they are his daughters.”*

- Barriers to end the relationship

Some women explained that the fact of being pregnant made it harder and more complicated to abandon the life project that they were building together with their partners. As Woman 6 said, “*the problem of motherhood, motherhood as a problem*.”

Woman 2 explained it in greater detail: ending the relationship at that time meant abandoning her entire life project:

Woman 2: *“I think another idea we would all share is that, when you are building a life project with somebody, breaking up at that time is not only breaking up with your partner; you break your structure, your project, your idea. And I think that is psychologically the hardest.”*

In fact, Woman 2 explained how sometimes the partner is aware that pregnancy can make it difficult for the woman to end the relationship and may use this to increase his control over her:

Woman 2: *“I also think, I wonder if you agree, in my experience, when the abuser wants to have a child it’s a way to keep you there (…). And afterwards, in retrospect, it’s clear that it is also a way of always keeping you there.”*

All the women agreed that ending the relationship or seeking help from their social network becomes even more complicated when certain feelings such as shame and guilt come into play. Blaming themselves along with the fear of how other people will react was considered a major barrier for ending an abusive situation. In some cases, participants reported how they decided not to tell anybody for this reason, even though they were aware of their situation:

Woman 1: *“Well, in my case, I was very embarrassed also when I started to realize it, I didn’t tell, I didn’t tell anybody.”*

Woman 2: *“Because it’s like talking badly about yourself.”*

Blame was also mentioned as a means of control by the partner, so that everything negative that happens is the woman’s fault. This further complicates the search for help, reduces the real perception of the abusive situation and affects the wellbeing and autonomy of women:

Woman 3: *“Oh yes, because everything is always your fault, at least for my husband. I was to blame for everything: for the rain, for him losing his job, for everything.”*

Another aspect women reported as being of great concern was the lack of economic independence, which becomes even more relevant when there are children to be considered. This makes any possible action to end the relationship very difficult.

Woman 4: *“The economic issue is key (general agreement). If you don’t have a job, you don’t have any money, you see yourself like …”*

Woman 2: *“And it’s no longer just you, it’s also the children.”*

### Video counseling intervention: key aspects

Specifically, regarding the possibility of conducting psychological video counseling sessions with pregnant women in an IPVAW situation, the women were asked what content they considered essential to address, and what came up when thinking about these sessions. This last question caused debate about the feasibility, safety and barriers of this type of counseling.

#### Contents

When asked about what contents a video counseling session should include, four categories were identified as being of particular relevance: the lack of awareness of IPVAW, self-esteem and fears, and legal advice.

- Lack of awareness of IPVAW

Awareness of the IPVAW situation is an aspect that all women defined as very complicated but also as necessary both to eliminate the idea that they are responsible for what is happening to them and to consider how to take action against the abuse. All participants agreed about the importance of this aspect and mentioned it repeatedly, considering that it is essential to address it in the sessions.

Woman 1: *"I think it is key [for the woman to be aware of her situation]. That’s my opinion. Because you don’t realize most things that are happening."*

Woman 5: * "It’s very important to understand very clearly what is happening, because doubt is what prevents you from acting (general agreement). Thinking that the situation is your fault. It is very important to know that this is real, that it is happening and that you need to take a step forward."*

Participants also highlighted that making these types of resources available can be useful to increase awareness, even among women who are offered video counseling and reject it. They consider that giving visibility to resources directed to women who may be experiencing IPVAW would help increase awareness of their situation.

Woman 2: *“I think that, in fact, if these things didn’t exist, it would take you longer to realize (general agreement). So, getting help in the end, I think the end of the story is positive, even if there are so many women who say, “No, I’m not being abused” or “I don’t want to deal with that, I’m afraid” and so on. But the more this becomes normal and the more it is done, the more helpful it will be for these women.”*

In relation to this lack of awareness, participants pointed out the importance of how to confront women with reality. According to the participants, telling the woman directly that she is in a situation of abuse and that she should leave is counterproductive and negative. Woman 4 explained the importance of approaching this topic properly, and also described how she experienced the moment when she was told that she should end the relationship:

Woman 4: * "And I think that making you aware, I mean, telling you some things. The first time I was asked “Have you been abused?”, No, I haven’t… no, no, no. Because I didn’t want to admit it and also I didn’t want to see myself as a victim. You need to gradually see it for yourself rather than have somebody tell you directly, you know?"*

Woman 4: * "I think it is counterproductive. For example, when people say, “You should leave him” if they say you should leave him directly it’s like … “Boom”."*

- Self-esteem and fears

Several women reported that they consider it a priority to address self-esteem and fears, a proposal supported by all the participants. This topic led to a dialogue between Woman 1 and Woman 2; they both stated that they had felt similar fears:

Woman 1: *“Self-esteem, for example. Removing all fears, because I was afraid of driving…”*

Woman 2: *“Yes, so was I.”*

Woman 1: (…) *“Addressing the issue of fears and also self-esteem, I think (general agreement).”*

- Legal advice

Along with fears, the concern about certain legal processes appeared repeatedly, especially those related to divorce and custody. The legal process, reporting the abuse and the rights of IPVAW victims were topics that women considered to be overlooked. They agreed that there is a need to address the lack of information on these issues and to provide resources and specialized support for this.

Woman 6: “*Legal advice (general agreement). Yes, because you find yourself… it doesn’t matter if you are cohabiting, single, or married, because it’s a child that is your child and his child after all, and if you really want to break that relationship, you need to think of how you should do it to become separated, regarding custody issues… Get some information about whether you should report the situation or not, if you are experiencing abuse, in short, you should know what is at your reach, because we hear some things. But I don’t know…”*

#### Video counseling

In addition to the contents that video counseling should include, a discussion was raised about the technical aspects that should be taken into account when offering this kind of eHealth strategy.

- Feasibility

Conducting the sessions in video conference format raised certain doubts among the participants. This type of communication has some requirements, as Woman 2 explained: “*Wanting to talk, having a safe and quiet place to talk, not running any risks, of course …”.*

Given that finding a place to conduct the sessions is one of the most complicated aspects, many of the suggestions focused on conducting them somewhere outside the home, perhaps taking advantage of a visit to the midwife, where a space could be provided for the session with the counselor. Once again, the figure of the midwife as a facilitator emerged.

Woman 2: *“I agree with her, with the excuse of something related to the pregnancy, I could go to a certain place that is not my home or anybody’s home and I have a space to talk, something like that…”*

Woman 4: *“In your sessions with the midwife for the checkup, she can say “should we have a more thorough examination”? And with that excuse you can have half an hour to talk with the counselor (general agreement).”*

One of the participants pointed out that concealing the sessions as routine care could serve as a cover-up explanation to give to the partner about where the woman is going:

Woman 6: *“It would be easier if it was in the sessions with the midwife, that is, if you went to see the midwife, as you said. It doesn’t matter if it is a video call or physical visit but, even as an excuse (general agreement) to be able to say where you are going… so that you can have some kind of control, but… it’s complicated.”*

For the women, privacy is an important factor to take into account, even if the sessions are not held at home, because the woman may not feel free to speak for fear that someone will overhear the conversation.

Woman 2: *“But even if he is somewhere else and you don’t want anybody to hear. I mean, it’s not only him; with him you run a huge risk, but it’s also that you don’t want other people to know.”*

If the sessions are conducted at home, a factor that can complicate the situation is the presence of children, as reported by Woman 6. Being alone is complicated in this situation, as the woman would also have to find someone to leave her children with during the sessions.

Woman 6: *“Yes, and we haven’t talked about that, but perhaps you are pregnant and have two children and if you’re home alone with the two children it’s not easy to find a private space…*”

- Safety

Another issue that arose from the previous question was that of safety, that is, the fear that the partner will find out that the woman is attending the sessions. In particular, in the case of one of the survivors, her partner was a computer technician, which would make it more difficult to use technological means for the intended purpose:

Woman 4: *“Him finding out. He had control over my mobile phone. He was a computer technician and used to reset my mobile phone when he could from a distance. He reset it, he asked me for my WhatsApp, my Facebook…”*

Another concern mentioned by the participants was that, if the sessions were conducted at home, the partner could appear during the course of one. To solve this safety problem, they proposed to agree some type of signal with the counsellor, which would allow women to change the conversation and continue the session as if it was a pregnancy follow-up.

Woman 3: *“I think that perhaps if he is informed that now, I don’t know, for various reasons, there’s a new thing and midwives or family doctors do their follow-up that way, perhaps if you’re talking to this person you can continue talking as if you were talking to your midwife or your family doctor, your gynecologist, and the other person, your counselor … would know that, well, the conversation changed a bit, and know what is happening.”*

- Barriers

When participants were asked the first thing that came to mind about the possibility of speaking with a counsellor by video conference, certain barriers arose. Woman 2 said she would feel “coldness”: “*Yes, because I would be talking to a stranger through a camera and I don't know if she would give me much help…”.*

When asked about which topics would be difficult for them to address in these video-sessions, there was agreement among the participants that the episodes that are most violent or related to sexual intimacy would be the most difficult to share.

Woman 1: *“I found it very hard to describe the most aggressive episodes, for example, when he took our 8-month-old baby boy and went missing all day from 8 a.m. to 1 in the morning, with no bottles or anything, that is, in order to hurt me … That was hard for me. And then when Elena (the counselor) asked me questions about intimacy in the couple… that was terrible, it killed me, it was very hard for me to talk, she had to guide me a bit.”*

### Contributions for the safety planning app

Finally, the group discussed the opinion of women on the use of an application for the development of safety plans, including safety while using it and the contents it could include.

#### Safety while using the app

- Password and masking

When addressing the possible options to ensure safe use of the app, there were proposals to use one or two passwords so that the partner cannot easily access it:

Woman 5: *“That is, you access it with a fingerprint password, but inside you must access from another point because imagine if he asks, “open that app and tell me what that is”. And you would have to do it. When you open it, you should find something, I don’t know, something that is completely unrelated…”*

The group discussed the appropriateness of the application being masked so as not to attract the partner’s attention. Women proposed to mask the app by taking advantage of the pregnancy situation, making it look like an app with information about the different pregnancy stages.

Woman 6: *“A game for babies, for children, something about motherhood, since we are into that.”*

Woman 3: *“As you said it is camouflaged to look like another app, it could be another app like “First month of pregnancy, you have to do such and such thing, on another month you have to do something else”. And then you have the option of looking at pregnancy instructions or opening the app…”*

- “Quick leave” button

In addition, women considered it useful to include a “quick leave” button in the app, in case the partner appears while they are using it. They claimed that this would make them feel safer when using it.

Woman 4: *“And also if you can do things like… I find it very useful when you are using the laptop and open a screen with landscapes and, if you’re looking at something and he comes, you just click on the button and the screen pops up. That way if you are looking at something else he doesn’t realize. For example, perhaps you are looking at your plan and if he comes close or you see him come close you do that and the motherhood page appears, you know?”*

#### App resources

After showing the participants different features that safety planning apps commonly have, the group discussed how such features could help women who experience IPVAW during pregnancy and how they could be improved.

- Contacts

One of the sections that was proposed to include in the app was *Contacts*, with relevant phone numbers (e.g., emergencies, specific IPVAW resources, and relatives/friends). All the participants mentioned that they imagined themselves using this section, and one of them added the following:

Woman 1: *“It’s great. I wish I’d had that.”*

In this section, women were asked whether they thought it would be appropriate or not to include a confirmation button when calling the emergency telephone number. All the participants agreed that it would be better to eliminate it as they considered the speed with which they could call in an emergency situation was more important than the drawback of having to apologize if they called unintentionally.

- Peer stories

Another section that was proposed for inclusion was stories of other women who overcame the abuse. In this section, the stories of IPVAW survivors are shared, so that women who use the app can feel that they are not alone and that other women in the same situation were able to overcome it. Participants considered that it was not a good resource. They argued that hearing stories about other in their situation did not help them, referring to the support group they attended:

Woman 2: *“That didn’t do me any good. It didn’t because I was focused on my own issues, you know? The intention is great because you are trying to help, but listening just for the sake of listening to stories like yours when you already have your own …”*

Instead of peer stories, women suggested including a chat where they can talk to other women, which could be more interesting and positive for them.

Woman 6: *“Like a chat between friends, you know? At times, for example, there may be 4 women connected, and it should be anonymous. Four women are connected: “what’s the matter? Well, I’m here, can you give me some advice?”*

Woman 2: *“That sounds like a good idea to me, because it gives you some anonymous company and it might be good.”*

- Mood rating

Another proposal was to include a section where women can rate their mood each day, but it generated rejection among all participants. Women argued that the rating would always be negative and this would have a negative effect.

Woman 6: *“That’s a bit depressing, isn’t it?”*[Laughs]

Woman 1: *“The way I see it, with the huge problem you have and the anxiety you have, everything is going to be “negative, negative, negative”.”*

Woman 6: *“It should be something to raise your spirits, not to realize how bad you’re feeling.”*

- Information on how IPVAW can affect pregnancy

The proposal to include information on how the abusive relationship can affect the pregnancy and the baby was also generally rejected, and women proposed to focus instead on positive content or advice from professionals. They proposed to introduce contents that can help them manage certain situations: for example, techniques for managing stress and fear. In general, they preferred the inclusion of positive contents.

Woman 1: *“As I said, something positive so that when I’m with it* [the app] *I can look at it and say, “Look, this makes me laugh”.”*

Woman 2: *“I find it much more interesting to learn relaxation techniques, how to breathe well, to relax. Something that helps you calm down.”*

Woman 6: *“Some advice, for example: “What to do after a situation of violence”, you know? That is, as counselors, therapists, whatever. If, when you are scared, you can read a paragraph written by a professional providing advice like: “When you are scared after a situation of violence” or “When you are scared for your children”.”*

- Safety plan

Regarding the development of a safety plan in the app, participants considered that it may be useful, especially the process of creating it, because it clarifies the strategies that women may have when faced with a dangerous situation. They hardly see themselves using the application at the time of a risk scenario, but believe that having previously thought about these strategies can have a positive effect when responding in each context.

Woman 6: *“Also, to practice using these resources, you internalize them better and gradually get better at using them … indeed.* [You learn] *which ones are automatic, which ones are appropriate, which ones are not. For example, screaming helps me. Keeping quiet doesn’t, so you include it in your everyday life. It’s an analysis rather than something you are going to do at a given time. That is, a self-knowledge exercise.”*

Woman 4: *“It’s a plan, in my opinion, for example, at that moment I would have liked to know: first call the police, then call so and so, then call the lawyer, then call… I don’t know, those steps. Because I was confused and thought: “What should I do? Where should I go first?”.”*

Women also considered it positive to have emergency contacts so that they could call quickly as part of the safety plan if necessary.

## Discussion

The main objective of the present study was to identify the needs, concerns and preferences of survivors about the use of eHealth strategies to counsel and empower pregnant victims of IPVAW in antenatal care.

### Main findings

The focus group session conducted allowed participating survivors to share diverse and valuable experiences, opinions and suggestions. To our knowledge, this is the first study that addresses the use of eHealth strategies to address IPVAW during pregnancy with survivors. Relevant considerations about pregnant women who experience IPVAW were provided by the participants. According to them, offering video counseling to pregnant women is feasible, especially if some aspects regarding the contents and safety of the intervention are taken into account. Participants also discussed the usefulness of some of the typical features of safety planning apps and how to improve them for use by pregnant women who experience IPVAW.

Based on participants’ input, while addressing pregnant women that might be experiencing IPVAW, health care professionals are essential to detect violence but also to help victims during the process of ending an abusive relationship. Midwives play a key role in detecting violence and providing support at early stages, and training them in this issue can be of great interest [35]. Indeed, informants regarded the midwife consultation as the appropriate place to be introduced to eHealth strategies and counseling, acting as a facilitator of the entire process. The context of the midwife consultation is adequate for this purpose for several reasons. First, midwives have frequent contact with women during pregnancy, allowing them to develop a relationship in which women are more likely to talk to the midwife, compared to other healthcare professionals who attend women from time to time. In addition, pregnancy is a very special and intimate stage in a woman’s life. In this sense, the possibility of talking and sharing with the midwife certain sensitive issues and concerns related to pregnancy may also result in a more trusting and closer relationship with her. Having established this bond with the midwife, women may feel more comfortable and willing to discuss other sensitive topics such as violence and the use of eHealth strategies. Moreover, midwives can take advantage of this first step of IPVAW screening in health care centers to activate existing protocols and provide women with a referral network to different healthcare professionals. Participants considered it appropriate and necessary to be screened using eHealth strategies for IPVAW during pregnancy in primary care settings, which adds up to previous studies with conventional screening strategies [36]. Counselors were also described as an important figure in the process of ending the abusive relationship during pregnancy. Although previous studies in non-pregnant women also point to the relevance of health care providers in IPVAW situations [37], the relevance of midwives was particularly highlighted by pregnant women.

Among the concerns of pregnant women who experienced IPVAW about the use of eHealth strategies, some of the most cited ones were the safety of their children, the consequences of ending the relationship, the shame and guilt, and economic problems. Previous literature has addressed the relevance of their children for victims of IPVAW [37] and the critical role that pregnancy has for making relevant life-changing choices [38] since children change women’s perspective of the relationship and can encourage them to end the relationship or seek help. However, some participants perceived that pregnancy is a period in which it is harder to end the relationship since they felt that it could be harmful to their children. Fear of ending a relationship is a common issue for women who experience IPVAW [37]. According to our results, this fear is increased when the woman is pregnant, as it raises new concerns about the future of the child. Feelings of shame and guilt are some of the biggest barriers for non-pregnant women to seek help and support [39] when experiencing IPVAW. This is aggravated in pregnant women, since they feel that by staying in an abusive relationship, they are putting their children at risk. Economic problems are another main concern for pregnant women who experience IPVAW, since the needs of the child are also at stake, thus increasing the helplessness of women. Economic independence is crucial in the recovery from violence [40] and becomes a bigger issue during pregnancy.

Online interventions that include counseling have been previously implemented in non-pregnant women who experience IPVAW, with mixed results regarding its efficacy [24, 26]. In our focus group, video counseling was seen as a viable tool that was generally accepted as a potential helpful tool for pregnant women who experience IPVAW. Video counseling interventions should be directed towards some of the main concerns of victims, such as self-esteem, fear and awareness. These contents fit with the proposals of the Psychosocial Readiness Model, [41] which describes several internal and external factors that influence the process of change of IPVAW victims. The internal factors are awareness, perceived support and self-efficacy. In this regard, some proposals made by the participants coincided with some of the main applications of the model: providing information about IPVAW and healthy relationships to raise awareness, helping the women to identify supportive people, and promoting their empowerment. Regarding the external factors, the model highlights two important factors: the role of interpersonal interactions and situational events. Thus, midwives can provide positive interpersonal interactions and provide support to victims by listening to them and caring for their wellbeing. They can also address the external factors by referring women to specialized resources and by providing information about resources for dealing with housing, legal, employment, financial and other situational issues that may be of concern to women.

Another model that is widely used in healing and empowering victims of IPVAW that also coincided with some of the content proposed by participants is Dutton’s Empowerment Model [42], which explains that these interventions should aim at one or more of the following main goals: protection, enhancing choice making and problem solving, and healing post-traumatic reactions. Among these, protection must always remain a first priority, which is related to the multiple references to safety made by women throughout their contributions.

For video counseling to be a feasible tool, it is essential to make it safe and adapted to pregnant women’s needs. Our results suggest that allowing pregnant women to choose the time and place for the counseling and developing safety signals is a good option to increase their safety. It is important to take into account that video counseling interventions might raise some initial barriers for pregnant women – such as feelings of shame or seeing the situation as being cold and impersonal – that should be addressed by health care providers, especially in countries where there is no tradition of the use of eHealth strategies. Yet, use of these strategies is becoming more common since the onset of the COVID-19 pandemic.

Based on our results, safety planning apps are considered a useful resource for pregnant women who experience IPVAW, provided that they have proper safety features and contents appropriate to the situation. Regarding safety, the addition of a “quick leave” button and masking the app on the phone are some of the most important strategies to take into account when designing safety planning apps for pregnant women. Participants considered that adding a feature that allows users to preset contacts for quick calls to relatives and emergency numbers is essential, as well as including a map of resources that women can access at any moment. Some features that women did not consider useful to include in safety planning apps were peer stories and mood rating, which were not seen as helpful. Safety planning apps have been effectively implemented with non-pregnant women [43, 44], facilitating safety decision making [45] and reducing IPVAW exposure^23^. However, evidence on the implementation of safety planning apps in pregnant women is lacking, and these results can serve as a first step toward designing future studies on this topic.

### Recommendations for practice and research

The findings of this study should be taken into account when designing eHealth interventions for women who experience IPVAW during their pregnancy. Special consideration should be put into the crucial role of midwives as a first step in identifying intimate partner violence and as facilitators to introduce relevant eHealth strategies directed towards IPVAW, since they have the right context to build a trusting relationship and are able to keep a continuous contact with women during pregnancy. To this end, midwives should receive specific professional training related to the most common signs and symptoms of intimate partner violence, as well as communication tips to overcome women's reluctance to disclose abuse.

When trying to implement a video counseling session, it is key to ensure the safety of women and to adapt the intervention and its contents to the needs of pregnant women. When implementing safety planning apps, it is important to mask the app in the device and to include “quick leave” buttons for safer use. There is a lack of research on the use of eHealth strategies for pregnant women who experience IPVAW, and future randomized controlled trials should provide more insight on the implementation and effectiveness of such strategies in primary medical care. Additionally, it would be of great interest to address how different cultural contexts may influence the needs and concerns of pregnant women when eHealth interventions are offered. We also recommend input from patient and public involvement to develop new screening tools and interventions directed towards IPVAW in order to make them safe and better suited for women’s needs.

### Limitations

The main limitation of the present study was its small sample size. A larger sample size would have provided not only more consensus on the subjects addressed during the focus groups but could also have raised additional points that were not present in this study. Additionally, we recruited women of similar age, nationality and educational attainment. Including women from different socioeconomic and cultural contexts status would also provide relevant information.

## Conclusion

Taking the opportunity to listen to the proposals and opinions of IPVAW survivors allowed us to outline some key elements and factors in the development of eHealth interventions. In general, participants considered that the use of these tools can be very useful in the management of IPV during pregnancy if their suggestions are taken into account. Conducting focus group sessions would allow for a more in-depth understanding of their experiences and needs that could be very beneficial when developing these types of tools.

## Data Availability

The datasets generated and analyzed during the current study are not publicly available to protect participants privacy, but are available from the corresponding author on reasonable request.
